# Mechanism of *E*. *coli* Inactivation by Direct-in-liquid Electrical Discharge Plasma in Low Conductivity Solutions

**DOI:** 10.1038/s41598-019-38838-7

**Published:** 2019-02-20

**Authors:** P. Estifaee, X. Su, S. K. Yannam, S. Rogers, S. Mededovic Thagard

**Affiliations:** 1 Clarkson University, Department of Chemical and Biomolecular Engineering, 8 Clarkson Avenue, Potsdam, NY 13699 USA; 2 Department of Civil and Environmental Engineering, Clarkson University, Potsdam, NY 13699-5710 USA

## Abstract

This work investigates and reveals the main mechanism(s) responsible for inactivation of *E*. *coli* by in-liquid pulsed electrical discharge plasma in low conductivity solutions. Experiments were designed and performed to explore the effects of plasma-emitted UV light, oxidative radicals, and electric field on *E*. *coli* inactivation curves, rate of DNA leakage and visual appearance of the treated microorganisms. Results showed that electric field had the main role in inactivation; scanning electron microscopy images revealed that both plasma and the isolated electric field result in extensive cell wall disruptions. While this damage in the case of plasma treatment was extensive and distributed randomly along the envelope, the electric field-induced damage resulted in disruption primarily at the poles of the bacterial rods. Subsequent experiments conducted with an oxidative radical scavenger suggested that plasma-generated radicals do not contribute directly to the inactivation but assist in cell wall deterioration and extension of the ruptures first generated by the electric field.

## Introduction

There has been growing interest in plasma technology for water purification, surface modification and biomedicine, as well as other potential uses. The effectiveness of plasma in sterilization and inactivation of bacteria and other pathogens has also been investigated for a wide range of applications in agriculture, medicine, and food industry arenas. Atmospheric pressure plasmas that operate as dielectric barrier discharges and plasma jets have been successfully used to inactivate both Gram positive and Gram negative bacteria, biofilm formers, bacterial spores, fungal spores, yeasts, parasites, and viruses achieving reductions as high as five logs^1^. In fact, several of these atmospheric pressure devices have been commercialized^2,3^. The mechanism of their sterilization has been attributed to the UV light-induced destruction of the genetic material^4^, attack of oxygenated species on the cell DNA, fatty acids, and amino acids (i.e., etching), and direct volatilization of the microorganism material^4,5^. Electrostatic forces also may contribute to the rupture of cell walls^6,7^.

Electrical discharges formed directly in a liquid that generate joule per pulse-range plasma have been shown to inactivate *Escherichia coli*, *Staphylococcus aureus*, *Salmonella enterica*, *Microcystis aeruginosa*, *Pseudomonas putida*, *Bacillus subtilis*, and *Legionella pneumophila*, among other microorganisms^8–13^. Bacteria have also been inactivated by higher kilojoule per pulse discharges and by use of different discharge electrode materials including copper, nickel, chromium, tungsten, and stainless steel^14,15^.

The mechanism of sterilization by plasmas formed within a liquid is less understood than that of gas plasmas, primarily due to differences in the discharge physics and chemistry within and immediately surrounding the plasma channel. Like gas plasmas, in-liquid plasmas are powerful sources of short-lived active radicals (OH, H, HO_2_), electrons, ozone, hydrogen peroxide, and UV light^16,17^. Physical effects such as shockwave formation and the presence of strong electric field are characteristic for discharges generated directly in a liquid, especially for arc and spark discharges^15,18–20^. In general, sterilization effects of liquid plasmas have been attributed to combinations of chemical (hydrogen peroxide and reactive oxidative species), physical (shockwaves and UV) and electrical effects^11,16,21–23^. Efforts have been made to decouple the individual physical and chemical contributions to proposed inactivation mechanisms, but with contradictory and generally inconclusive results. For high energy spark and arc discharges, plasma-generated UV light and shockwaves are often proposed as the dominant mechanisms of inactivation^10^. For low energy streamer discharges, the combination of UV light and chemical agents is often assumed^8,24,25^. The aim of this study was to investigate the contributions of UV light, generated free radicals and electric field to bacterial inactivation during low energy streamer direct-in-water pulsed electrical discharge plasma treatment. The experiments were conducted on a low conductivity (100 µS/cm) suspension of *Escherichia coli*, which was used as a model organism owing to its fast growth rate and use in other published studies facilitating comparisons. The study has been conducted in an air-free environment and as such does not assume the presence of reactive nitrogen species (RNS) nor discusses the RNS-based mechanisms of inactivation. Identification of the primary mechanism of inactivation of the in-liquid plasma treatment would allow for informed modifications in plasma reactor design and further improvements in the efficiency of this technology for biological, medicinal, and agricultural applications.

## Materials and Methods

### Bacterial Culture

*E*. *coli* ATCC 700891 (American Type Culture Collection, Manassas, VA) was used for all experiments, and maintained as per manufacturer’s instructions. Briefly, cell suspensions were prepared fresh for each experiment in 100 mL tryptic soy broth (TSB, BactoTM, Maryland, US), and incubated overnight at 37 °C. Cells were harvested by centrifugation at 4000 × g for 8 min and washed three times in phosphate-buffered saline (PBS, pH = 7.4) before use.

### Plasma Reactor

**Figure 1:**
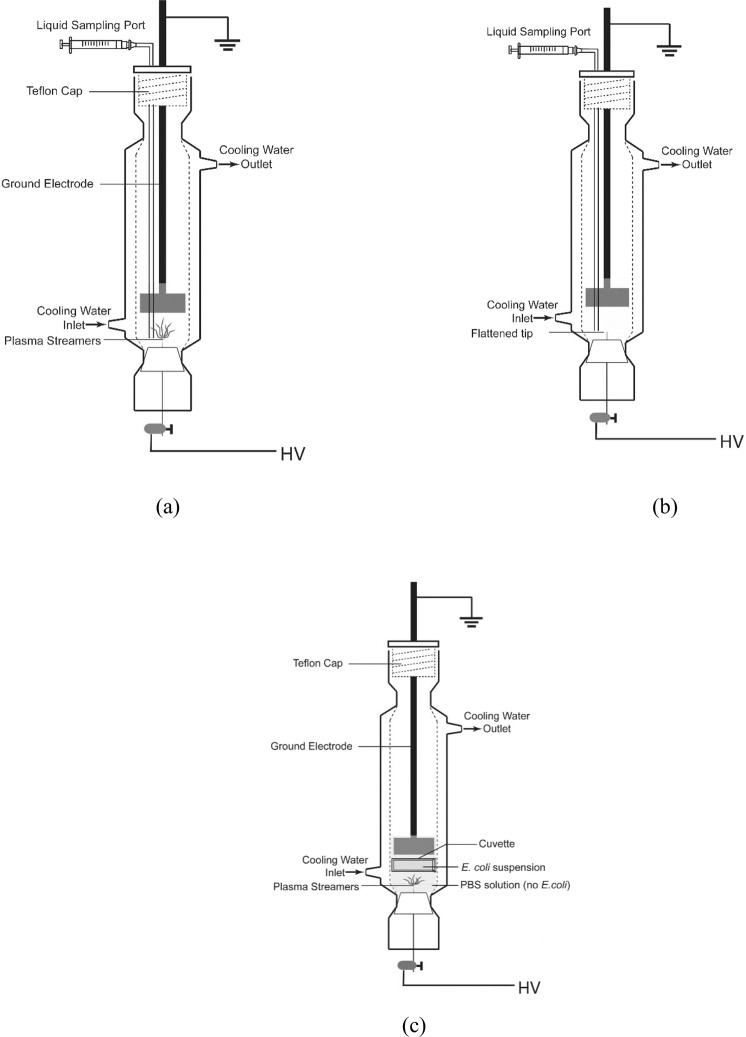
Schematic of (**a**) the plasma reactor, (**b**) the PPEF reactor and (**c**) UV experiment plasma reactor set up used in this study.

The batch reactor used in the study is shown in Fig. 1a. Briefly, a 27 cm long jacketed glass vessel 4.3 cm in diameter was fitted with Teflon caps on both ends to facilitate insertion of electrodes and liquid sampling. The high voltage (HV) electrode was a NiCr wire (diameter = 0.8 mm). The ground electrode was a stainless-steel disc (diameter = 3 cm). The electrodes were immersed in solution and separated by an electrode gap of 2.5 cm. The electrical circuit used to create plasma was previously described^12^. A high voltage DC power supply was used to charge a 0.75 nF load capacitor to + 17 kV; the stored charge was discharged into the plasma reactor via a rotating spark gap. The frequency of the discharge was determined by the rotation speed of the spark gap, which was adjusted to 60 Hz (~0.21 J/pulse). The voltage and current during the treatment were recorded by the Tektronix P6015A high voltage probe and the Tektronix P6021 current probe connected to a Tektronix TDS 3032 C oscilloscope.

A “pulsed plasma electric field (PPEF)” treatment was used for some experiments of this study to isolate the effect of the electric field from that of UV light, free radicals, and the shockwaves produced when plasma is created in water. To create this condition, as shown in Fig. 1b, the reactor was modified by flattening the tip of the high voltage electrode to suppress plasma formation with applied voltage.

To examine the contribution of UV to inactivation of *E*. *coli* during plasma treatment, 5 mL of *E*. *coli* suspended in PBS was isolated within a quartz cuvette and suspended/fixed between the ground and HV electrodes in the plasma reactor, as shown in Fig. 1c. The cuvette isolated the effects of UV from the electric field, free radicals produced in solution by the plasma treatment, and shockwaves that may be generated during the discharge.

**Figure 2:**
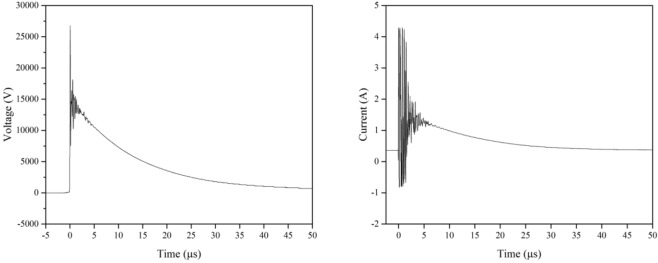
Voltage and current waveforms for direct-in-liquid discharge.

Representative voltage and current waveforms for experiments in which plasma was formed are shown in Fig. 2. The shapes and magnitudes of both waveforms were identical for the PPEF experiment (waveforms not shown). Solution temperature for plasma, UV and PPEF experiments was maintained at 20 °C with cooling water. The content of the reactor was not mixed, apart from plasma-induced mixing.

### Plasma Experiments

All experiments were conducted in triplicate. The reaction mixture (65 mL) contained *E*. *coli* (initial concentration of ~10^7^ CFU/mL) in PBS (0.1 mM, pH = 7.4) with electrical conductivity adjusted to 100 µS/cm (2.1 mM NaCl, 0.15 mM phosphate buffer, and 0.045 mM KCl). To prepare the reaction mixture, initial concentrations of *E*. *coli* were adjusted using sterile PBS and a UV-Vis spectrophotometer (Shimadzu UV-1800, Shimadzu, Japan). Absorbance was read at 600 nm and compared to a standard curve generated in our laboratory to achieve the desired initial concentration. If necessary, the electrical conductivity was re-adjusted to 100 µS/cm using sterile, deionized water or PBS. Initial concentrations of *E*. *coli* were verified by triplicate dilution plate counts (in sterile deionized water) on tryptic soy agar (TSA, ThermoFisher, US; incubation at 37 °C for 24 hours).

Inactivation of *E*. *coli* was measured during the time course of plasma treatment by dilution plate counting on TSA as described above. Briefly, total treatment times were 12 minutes. Every two minutes during treatment, ~1 mL sample was withdrawn from the reactor to facilitate measurement of bacterial decimation. Subsamples were also retained for scanning electron microscopy (SEM) and tests for DNA leakage as described below.

To investigate the contribution of free radicals to inactivation of *E*. *coli*, caffeine (a free radical scavenger) was used in the reaction mixture. In one set of experiments, 0.2 M caffeine solution was used, after which PBS was added to adjust the total solution conductivity to 100 µS/cm.

### Hydrogen Peroxide Measurement

The concentration of H_2_O_2_ was determined colorimetrically, using the reaction between H_2_O_2_ and titanium(IV) sulfate (159.93 M, ~15 wt% in dilute sulfuric acid, 99.99%, Sigma-Aldrich) test reagent and measuring the absorbance of the resulting yellow peroxotitanium complex [Ti(O_2_)OH(H_2_O)_3_]^+^ at 410 nm with a UV-Vis spectrophotometer. All experiments were repeated in triplicate.

### Scanning Electron Microscopy

Morphological changes in *E*. *coli* exposed to plasma were investigated using scanning electron microscopy as described by Dana Ziuzina *et al*.^26^. Briefly, the aqueous solution following plasma treatment was centrifuged, and the supernatant discarded to obtain dense cell pellets for SEM analysis. Pellets of *E*. *coli* were resuspended in sodium cacodylate buffer (SCB) with 2.5% glutaraldehyde and placed in an ice bath for two hours. Fixed cells were then washed three times in SCB, and post-fixed in 1% osmium tetroxide at 4 °C for 2 h. The fixed cells were then again washed three times with SCB, followed by three washes with deionized water. Samples were dehydrated using a 50%, 70%, 80%, 90%, and 99.5% ethyl alcohol wash series (Sigma Aldrich, 200 proof, molecular biology grade). Next, each sample was freeze-dried (Dura Stop/Dura Dry freeze dryer, model#TDS2C0B50B0), fixed onto carbon tape, and sputter coated with gold particles for 2 minutes to avoid surface charging. A JEOL JSM-7400F Field Emission Electron Microscope was used to take images while operating at 1 kV.

### DNA Leakage

Plasma treatment may result in formation of pores (disruption) across bacterial cellular walls, resulting in leakage of cellular components. The rate of leakage of cellar components into the solution was estimated by measuring free DNA concentrations in the reaction fluid with time during plasma treatment. Free DNA concentrations were measured in 20 µL subsamples at each 2-minute interval using the Quant-iT™ High Sensitivity (HS) DNA Assay Kit and Qubit 2.0 fluorometer (Invitrogen, Grand Island, USA) as per the manufacturer’s instructions. This kit is accurate for concentrations ranging from 10 ng/µL to 100 ng/µL. No potential chemical interactions between the electrolytes/compounds (PBS and/or caffeine) and the DNA HS assay kit were observed in control assays.

### Statistical Analysis

Statistical analysis was performed with R 3.5.0 and RStudio. Comparison of parameter levels was done with one-way analysis of variance (ANOVA). Group comparison was done with post hoc test (Tukey’s honestly significant difference, HSD). A level of significance of *p* ≤ 0.05 was chosen for all statistical analyses.

## Results

**Figure 3:**
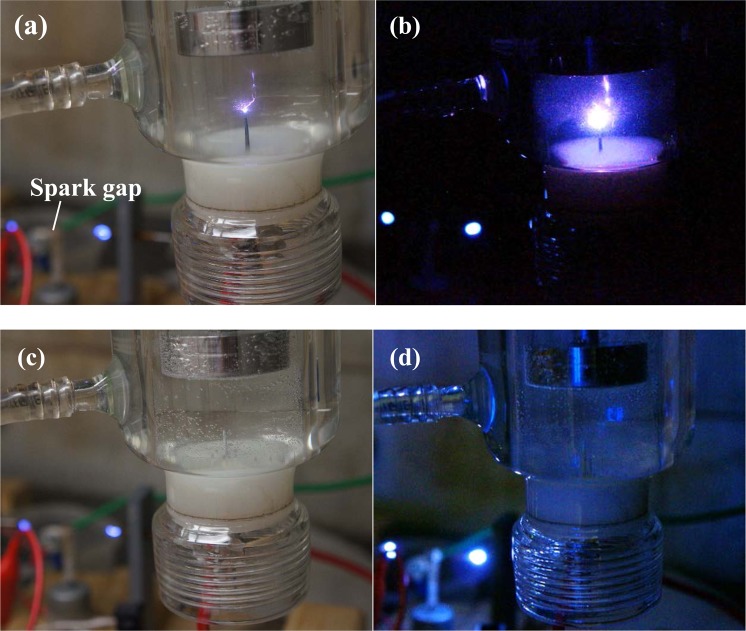
Pictures of the in-liquid electrical discharge plasma process in (**a**) light-on and (**b**) light-off conditions and PPEF process in (**c**) light on and (**d**) light off conditions.

**Figure 4:**
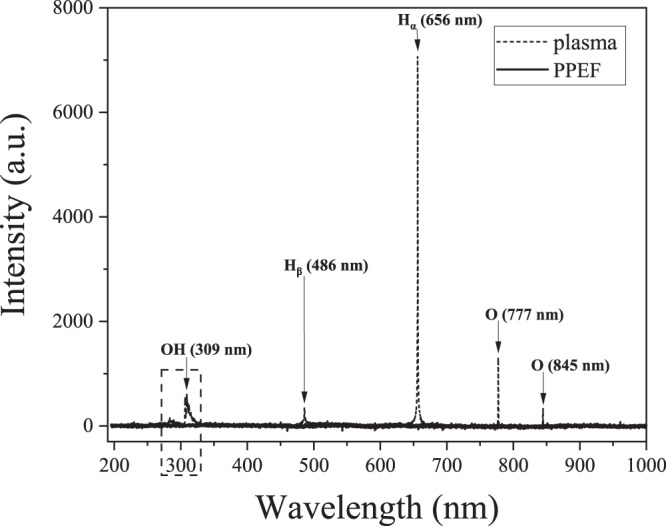
Optical emission spectrum of plasma and PPEF discharges in liquid.

The pictures of the plasma and PPEF reactors in operation are shown in Fig. 3 in both light-on and light-off conditions. As shown, plasma was absent for the PPEF treatment. The optical emission spectra for plasma and PPEF treatments are shown in Fig. 4. Expectedly, the radiation from electrical discharge plasma in water is dominated by the emission from OH radical (309 nm) and by the spectral lines of hydrogen (656 nm H_α_ and 486 H_β_) and oxygen (777 nm and 845 nm) atom. In contrast, short-lived species were not generated for the PPEF treatment.

**Figure 5:**
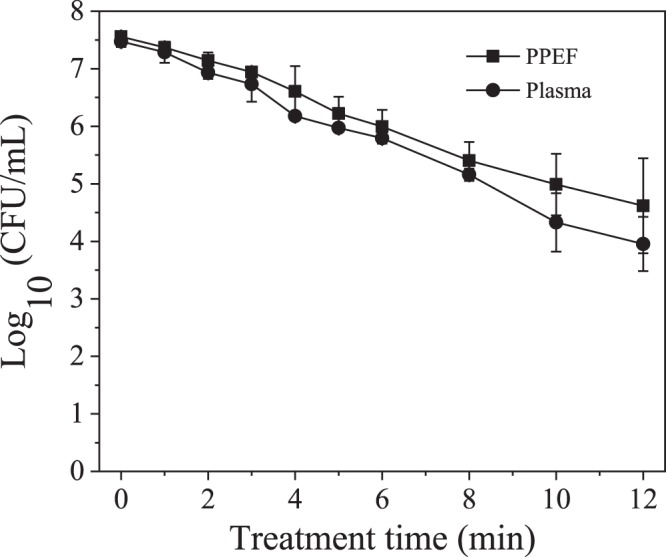
Inactivation of *E*. *coli* by direct-in-liquid electrical discharge plasma and pulsed plasma electric field (PPEF) treatments. pH and conductivity of the solution were 6.98 (99.4 μS/cm) before and 7.04 (104 μS/cm) after the plasma treatment. pH and conductivity of the solution were 7.02 (102 μS/cm) before and 7.24 (110 μS/cm) after the PPEF treatment.

**Figure 6:**
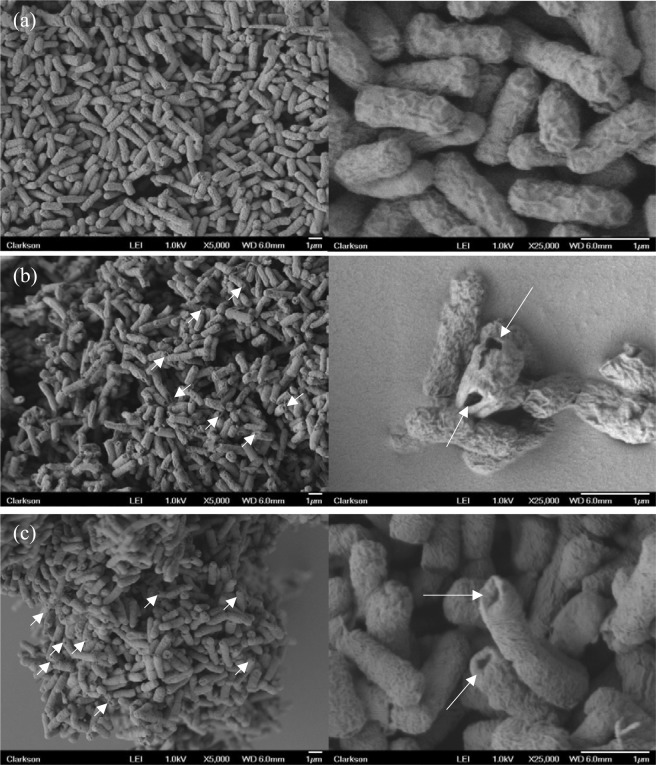
SEM images of (**a**) nontreated (control), (**b**) plasma treated, and (**c**) PPEF treated *E*. *coli* at 5000X and 25000X magnifications.

**Figure 7:**
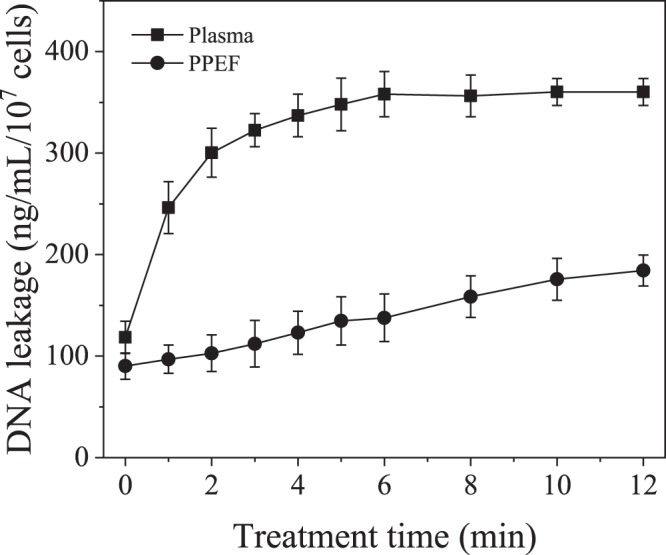
Normalized rate of DNA leakage from *E*. *coli* treated with plasma and PPEF.

Inactivation of *E*. *coli* when treated by plasma and PPEF is shown in Fig. 5. With plasma treatment a 3.5-log reduction was observed after 12 minutes compared to a 3-log reduction with PPEF. Statistical analysis indicates that the two treatments are not significantly different. SEM imaging was used to visualize damage to bacterial cell walls before and after the plasma and PPEF treatments (Fig. 6). As can be seen in Fig. 6, treatment with plasma and PPEF resulted in cell wall disruption. In the case of plasma treatment, the damage was throughout the cell walls. In contrast, PPEF treatment resulted in disruption primarily at the poles of the bacterial rods. Shown in Fig. 7 and confirmed by the results of one-way ANOVA and Tukey’s HSD test, the rate of DNA leakage was significantly greater in plasma versus PPEF treatments, consistent with the greater degree of cell wall damage observed in SEM images (Fig. 6).

**Figure 8:**
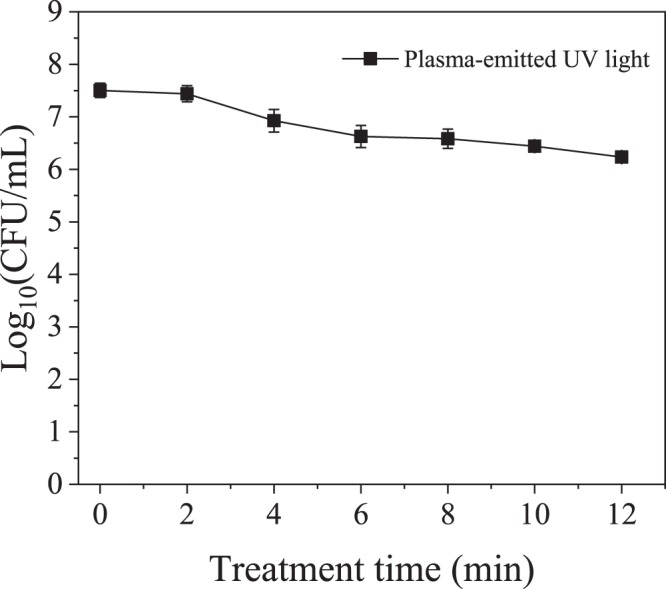
Inactivation of *E*. *coli* isolated within a quartz cuvette by plasma-emitted UV light.

**Figure 9:**
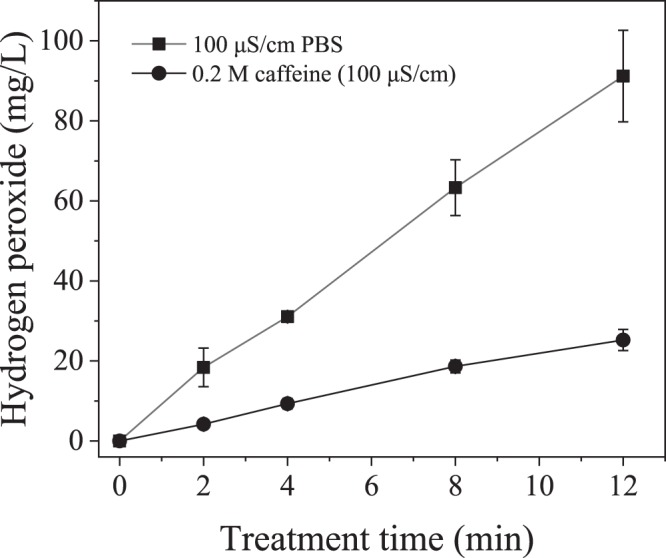
Hydrogen peroxide concentration with and without caffeine, an OH radical scavenger.

**Figure 10:**
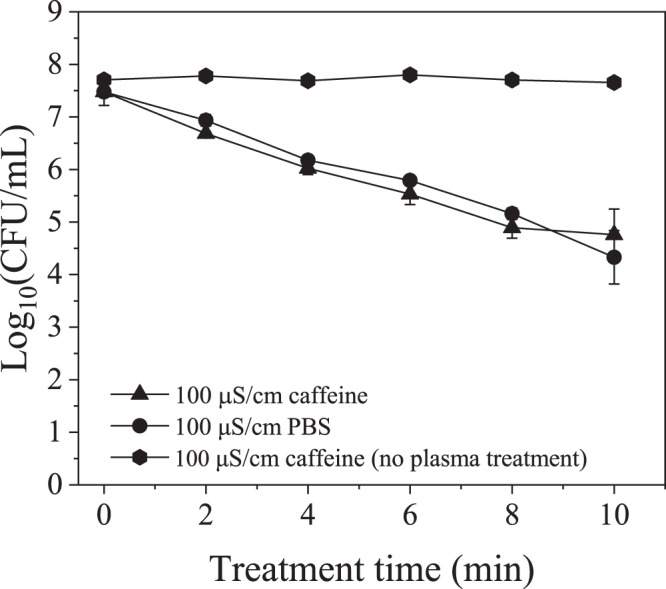
Effect of caffeine on inactivation of *E*. *coli* during plasma treatment.

**Figure 11:**
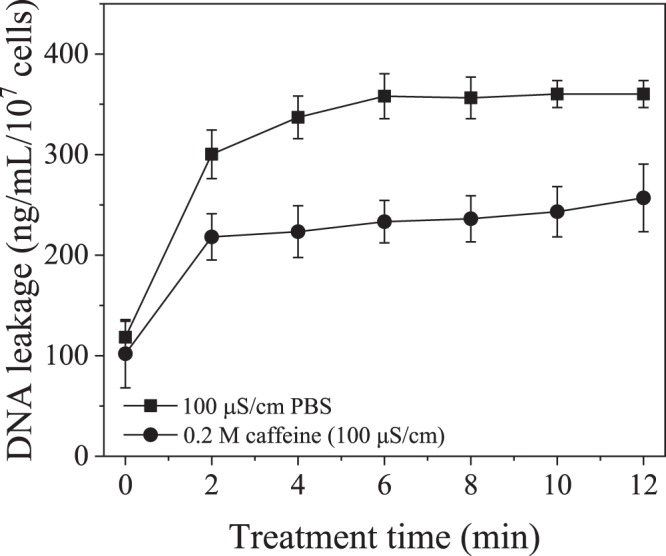
Normalized DNA leakage measurements of the plasma treated *E*. *coli* with caffeine and PBS as electrolytes used to adjust the solution conductivity.

**Figure 12:**
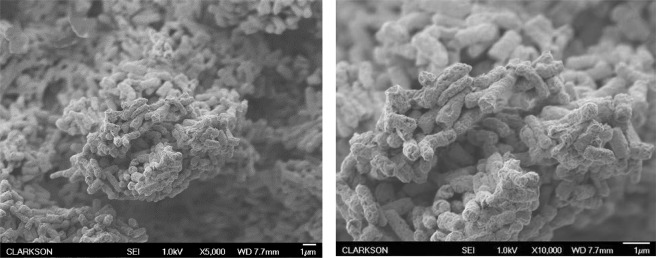
SEM images of *E*. *coli* following direct-in-liquid plasma treatment with a 0.2 M caffeine solution (conductivity 100 µS/cm) at 5000X and 10000X magnifications.

UV light produced by the plasma discharge had a negligible effect on inactivation of *E*. *coli* isolated in a quartz cuvette within the plasma reactor during plasma treatment in this study (Fig. 8). The OH radical scavenging capacity of caffeine was assessed by measuring the concentration of the OH radicals’ direct recombination product, hydrogen peroxide. As shown in Fig. 9, while the presence of caffeine resulted in more than 75% decrease in hydrogen peroxide concentration, it had no effect on the inactivation of *E*. *coli* during plasma treatment, indicating that the effect of free radicals generated during the discharge was minimal relative to the electric field effect (Fig. 10). For the applied concentration of caffeine, there were no interactions observed between caffeine and *E*. *coli* in the absence of plasma treatment. Figure 11 shows, and statistical analysis confirms, that the amount of DNA leakage for the caffeine case was lower than for the PBS case. SEM images of *E*. *coli* following direct-in-liquid plasma treatment with a 0.2 M caffeine solution (conductivity 100 µS/cm) were acquired to investigate the impact of free radicals on cell wall integrity (Fig. 12). In the presence of caffeine, the visible damage to cell walls was reduced (see Fig. 6b for comparison), in particular the appearance of holes at the poles of the bacterial rods.

## Discussion

Physics and chemistry of underwater electrical discharges have been studied extensively and several reviews on the subject already exist^27,28^. Optical emission spectroscopy measurements conducted on plasmas in water in this and other studies confirm the dissociation of water into OH, H and O excited reactive species^16,29^. Due to their short lifetimes, plasma-generated radicals recombine to form hydroperoxyl radicals and three stable products: hydrogen, oxygen and hydrogen peroxide^30^. Electrical discharge plasmas also produce physical effects such as (V)UV radiation and shockwaves, both of which are capable of influencing the chemical and biological processes surrounding the plasma channel, depending on the solution electrical conductivity and input energy^31^.

Several efforts have been made to understand the mechanism of bacteria inactivation by direct-in-water plasmas, in particular the roles of reactive oxidative species (ROS) and plasma-generated UV light. After identifying active species produced by streamer corona discharges in water, Sun *et al*. reported that OH radicals play a key role in bacterial inactivation^29^. Abu-Ghazala *et al*.^8^ also reported that OH radicals played a key role, while the contribution of UV to inactivation was negligible. Similar to studies on bacteria, OH and H radicals were reported to play no role in the inactivation of yeast cells^32^. Identical results were reported for free radicals and UV light contributions to inactivation of cyanobacteria where it was concluded that shockwaves were the primary mechanism of inactivation during a streamer-like discharge in water^33^. Finally, the extent to which the electric field generated by the power deposition into the plasma reactor contributes to inactivation of bacteria is unknown.

In this study, inactivation of *E*. *coli* was similar between plasma and PPEF conditions, indicating that electric field plays a dominant role in treatment (Fig. 5). Several other studies have shown that applying high voltage pulses results in disruption of bacterial cell walls^34–36^. Specific for plate-plate in-liquid discharge, up to 5% of the total inactivation has been attributed to the electric field^37^. Among different techniques applied to study inactivation of bacteria by electric field, fluorescence microscopy and atomic force microscopy provided visual demonstrations of electric pore formation^38^. By applying an external electric field to a cell, a transmembrane potential is induced that is proportional to the density of the electric field. When the transmembrane potential exceeds a critical value (~1 V for bimolecular lipid layer^39^), the membrane starts to break down. This process, known as electroporation, has several applications in gene transfer, gene therapy, cell molecular transport and electrofusion^40–43^. Pliquett *et al*.^44^ investigated the effect of applying high electric fields to cell membrane by molecular dynamics. Their modeling showed that for a bacillus-shaped cell wall such as *E*. *coli*, the poles of membrane tend to rupture and fragment more due to higher density of the electric field in these regions. As shown in Fig. 6c, in the case of PPEF, the ruptures were almost exclusively located on the poles of the bacteria which is consistent with the molecular dynamics modeling results discussed above.

The plasma-treated bacteria appear to have possibly larger polar holes per cell than the PPEF treated bacteria, as evidenced by the SEM images. In addition, the damage to the bacterial cell wall envelope is also visible for the plasma treatment. Since there is no available technique to quantitatively compare number or size of formed holes created due to plasma and PPEF treatments, DNA leakage was used as a surrogate (Fig. 7). DNA leakage was significantly greater for samples treated with plasma than PPEF, suggesting that plasma treatment results in a greater degree of cell wall disruption than PPEF treatment.

To examine whether greater cell wall deterioration was due to the attack of free radicals (which are absent in the PPEF treatment), measurements of DNA leakage were conducted for plasma-treated *E*. *coli* with PBS as the electrolyte (i.e., ion source) versus caffeine-containing PBS as the electrolyte. Caffeine has been used as a free radical scavenger in several other studies^45–47^. As shown in Fig. 11, almost 2 times more DNA was leaked without the scavenger. That significant difference in the extent of DNA leakage demonstrates that free radicals play a crucial role in the formation of holes and might be controlling their size. OH radicals produced from an electrical discharge in water are strong oxidants (E^0^ = 2.85 V/SHE) and can readily oxidize the phospholipid bilayer comprising the cell wall of Gram-negative bacteria such as *E*. *coli*. The product of the oxidation is malondialdehyde, and its formation during treatment of water with *S*. *typhimurium* has been measured in gas-liquid plasmas, verifying existence of this pathway^1,14,48,49^. Plasma also produces H radicals which can abstract a hydrogen atom from unsaturated carbon bonds of fatty acids causing lipid peroxidation^14,50^.

Electrical field appears to be responsible for rupturing the cell walls and allowing radicals (especially OH) to have greater access to the lipid tail of the outer phospholipid layer of the cell wall^44^. While plasma-generated radicals have no impact on kinetics of inactivation (Fig. 5), after the electric field has initiated hole formation, radicals have more access to the envelope, where they enlarge the holes resulting in higher DNA leakage. SEM images of samples from caffeine treatments (reduced concentration of free radicals) of this study (Fig. 12) showed fewer and smaller holes in cell walls as compared to the treated sample with no scavenger (see Fig. 6b). This result also may suggest that the holes found along the bacterial wall envelope are not actual holes but rather surface damage induced by ROS. The absence of a contribution by OH radicals to inactivation of microorganisms during a streamer discharge was confirmed by Lee *et al*.^51^ using tert-butanol as a radical scavenger.

The other possible mechanism in inactivation of *E*. *coli* by plasma treatment is UV light which is emitted from the plasma channel upon de-excitation of excited radical species^52^. The intensity of the plasma-emitted UV light has been shown to be a function of both solution electrical conductivity and applied voltage. High energy underwater discharges which generate spark and arc plasmas have been extremely successful in inactivating microorganisms, primarily through mechanisms involving UV light and shockwaves^10^ the latter of which are discussed later. The UV light has also been shown to inactivate microorganisms in lower energy (1–3 J/pulse) plasma treatments^24^. In that study, Lukes and coauthors demonstrated that the intensity of the 190–280 nm plasma-generated light in the conductivity range 100 µS/cm–500 µS/cm steeply increases with solution conductivity. In fact, the photon flux determined at 500 µS/cm was more than seven times higher than that for the 100 µS/cm solution at 21 kV applied voltage. The photon flux from the discharge (*J*) was shown to be proportional to discharge pulse mean power (P) according to the formula *J* = 44.33P^2^ ^11^. The authors estimated that at conductivity of 200 µS/cm and applied power at 95 W, up to 30% of the UV light emitted by plasma contributes to the overall inactivation. Similar result was obtained by Sun *et al*. though the discharge power and solution conductivity were not reported in the study^37^.

In this study, there was no evidence of significant contribution to bacterial inactivation by UV light. Most likely, the intensity of the photon flux emitted from a low power discharge plasma (13 W) at 100 μS/cm was insufficient to cause measurable DNA damage. This observation is consistent with results published by Abou-Gazala *et al*.^8^ and Lee *et al*.^51^.

Due to the complexity of shockwaves and the difficulty in quantifying and isolating them, no direct experiment of their effect was performed. Several studies evaluated possible damage introduced by shockwave to cells. Leighs *et al*.^53^ investigated the effect of shockwaves on *E*. *coli* cells. Examination of SEM images taken from *E*. *coli* that were damaged by shockwaves indicates that this type of cell wall damage is different than the damage observed in this research by either plasma or PPEF. After treatment with shockwaves, the *E*. *coli* cell wall is deformed and broken apart and no longer retains its rod shape. The similar type of damage of *E*. *coli* was observed by Lee *et al*.^20^ after an underwater pulsed arc discharge plasma treatment. The authors concluded that the primary mechanism of *E*. *coli* inactivation was shockwaves, which is not surprising considering the level of input energy required to create an arc discharge; the intensity of a shockwave directly depends on the applied power^54^ and is proportional to water conductivity^16,51^. In their underwater arc discharge study, the solution conductivity and the input power were 45 mS/cm (0.1 mS/cm for this study) and 10 MJ/pulse (0.21 J/pulse for this study) respectively. In this study, the general shape of the cell wall remains intact after treatment with either plasma or PPEF, and only some holes are formed in the cell wall.

Based on molecular dynamics simulations, Hu *et al*.^55^ demonstrated that shock waves can form pores on dipalmitoyl-phosphatidylcholine (DPPC) bilayer membrane. The application of an external electric field serves to keep the pores open, but also produce controlled expansion. This synergistic action of shockwaves and electric field is advantageous for many applications, however might not be applicable to prokaryotic cells. Unlike the mammalian cell, bacteria cell wall contains an outer membrane and peptidoglycan layer, therefore the response of bacteria cell towards the shockwaves and electric pulse might be different. An extreme example of this argument is a bacteria spore, which has a spore coat (keratin and other proteins) and cortex (peptidoglycan). According to Lamarche *et al*.^56^, no structural disorganization under transmission electron microscopy (TEM) is observed with spores treated by underwater electric arcs that in practice generate much stronger shockwaves than underwater streamer discharges. Nevertheless, if low power discharges in low conductivity solutions such as those used in this study produce shockwaves of measurable intensity, the influence of shockwaves on electroporation should not be ignored.

## Conclusions

The work conducted in this study provides a better understanding of the mechanism of *E*. *coli* inactivation in low power low conductivity solutions by direct-in-water electrical discharge plasma treatment. Experiments were performed to explore the effects of plasma-emitted UV light, oxidative radicals, and electric field on *E*. *coli* inactivation, rate of DNA leakage and visual appearance of the treated microorganisms. Through experiments, UV light was isolated and demonstrated to have no significant impact on *E*. *coli* inactivation. A plasma pulsed electric field reactor configuration was used to investigate the effect of electric field, the results of which were compared with plasma treated samples. The electric field played a major role in inactivation of *E*. *coli*. Using a free radical scavenger, it was demonstrated that free radicals had only a minor influence on bacterial inactivation; however, once *E*. *coli* were ruptured by the electrical field, free radicals contributed significantly to the cell wall deterioration and extension of the ruptures. A comparison of these results with those of other studies with identical electrode configuration reveals that there exists a close relationship between the inactivation mechanism and discharge power and solution conductivity. For low power (0.21 J/pulse) discharges in 100 µS/cm conductivity solution, the electric field is the dominant inactivation mechanism. To find the conditions at which the mode of mechanism of inactivation changes, future studies should make sure to include information on the solution electrical conductivity and energy per pulse alongside inactivation curves. The results of this study can be used towards optimization of an in-liquid plasma reactor for bacterial inactivation, and can be applied for the plasma treatment of other types of microorganisms.

## Data Availability

All data generated during this study are included in this published article.
